# To chat or bot to chat: Ethical issues with using chatbots in mental health

**DOI:** 10.1177/20552076231183542

**Published:** 2023-06-22

**Authors:** Simon Coghlan, Kobi Leins, Susie Sheldrick, Marc Cheong, Piers Gooding, Simon D'Alfonso

**Affiliations:** 1 School of Computing and Information Systems, 2281 The University of Melbourne; 2 Department of War Studies, King's College London; 3 Melbourne Law School, The University of Melbourne

**Keywords:** chatbots, artificial intelligence, ethics, mental health, data privacy

## Abstract

This paper presents a critical review of key ethical issues raised by the emergence of mental health chatbots. Chatbots use varying degrees of artificial intelligence and are increasingly deployed in many different domains including mental health. The technology may sometimes be beneficial, such as when it promotes access to mental health information and services. Yet, chatbots raise a variety of ethical concerns that are often magnified in people experiencing mental ill-health. These ethical challenges need to be appreciated and addressed throughout the technology pipeline. After identifying and examining four important ethical issues by means of a recognised ethical framework comprised of five key principles, the paper offers recommendations to guide chatbot designers, purveyers, researchers and mental health practitioners in the ethical creation and deployment of chatbots for mental health.

## Introduction

The rapid rise of chatbots in information and service provision by businesses, government agencies and non-profit groups^1^ has inevitably touched the domain of mental health. As benign as they may first appear, chatbots raise ethical issues. Mental health chatbots that offer information, advice and therapies have the potential to benefit patients and the general public, but they also have the capacity to harm vulnerable individuals and communities.^2^ Chatbots regarded as unethical may also damage the reputation of individuals and organisations who deploy them. Like some other digital tools, chatbots raise a range of specific ethical issues such as privacy, transparency, accuracy, safety and accountability.^3^ The importance of these ethical issues will only grow as chatbots get more sophisticated.

This paper provides a critical ethical overview of chatbots that provide information, advice and therapies to users in regard to mental health. It examines ethical issues for the design and deployment of these mental health chatbots and provides recommendations to guide their responsible development and use. The paper should be useful for chatbot designers, purveyers, researchers and mental health practitioners who seek a clear and solid framework for understanding and/or navigating the ethical issues that mental health chatbots create.

Chatbots have been defined as ‘any software application that engages in a dialog with a human using natural language’.^4^ Other terms for chatbots include dialogue agents, conversational agents and virtual assistants.^5^ Previous scholarly work has identified several ethical advantages and challenges of chatbots and similar technologies,^6,7^ and some works have offered ethical guidelines or frameworks relevant to mental health chatbots. For example, Wykes et al. examined ethical issues for a range of mental health technologies including health apps using the principles of privacy and data security, good development practices, feasibility and the health benefits of such technologies.^8^ Luxton et al.'s discussion of ethical considerations raised by intelligent machines for mental healthcare drew on work in robot and machine ethics as well as on professional ethical codes for mental health professionals.^9^ Meanwhile, Lederman et al. applied the classic four-principle ethical framework from medical ethics to a specific mental health intervention platform that some of those authors were developing.^2^

Our aim in this paper is to provide a critical review of ethical considerations regarding mental health chatbots in general. In contrast to other ethical discussions about chatbot-style technologies such as those just mentioned and others,^5,10,11^ we employ the recent five-principle framework developed within artificial intelligence (AI) ethics.^3^ This framework incorporates the classic and widely accepted four-principle framework from medical ethics,^12^ but it also adds to it by means of a fifth principle of ‘explicability’ which accommodates special features of intelligent digital technologies.

The paper runs as follows. We first provide some general background about the history of chatbot development (Section II). We then canvas the operation of chatbots in mental health and observe some benefits and challenges^13–15^ (III). Next we explain and then utilise an established and helpful ethical framework from medial and AI ethics (IV) to analyse core issues raised by mental health chatbots (V). Finally, we provide ethical recommendations that apply throughout the technology pipeline from inception to chatbot retirement (VI), with the aim of giving practical assistance to chatbot designers, mental health practitioners, researchers, etc. for ensuring that mental health chatbots are constructed and implemented in ethically defensible ways. Section VII summarises the discussion.

## Background to chatbots

Some general background about the history of chatbots will help in understanding their ethical implications in mental healthcare. Chatbots generally depend on some degree of natural language processing (NLP). NLP uses computing and statistical techniques, which these days often involves machine learning (ML), to ‘interpret’ human language and provide intelligible responses to human language input.^16^ The very first NLP computer program was created by Joseph Weizenbaum, a German–American computer scientist considered a father of modern AI. Named ELIZA, this ‘chatterbot’ simulated a Rogerian therapist (relating to the system of therapy developed by the psychologist Carl Rogers) by demonstrating ‘the responses of a non-directional psychotherapist in an initial psychiatric interview’.^17,18^ Weizenbaum aimed to show that ‘communication between man and machine was superficial’.^19^ Unexpectedly, however, Weizenbaum's assistant, though aware of the machine's purpose and limitations, began disclosing personal matters and forming a superficial relationship with ELIZA.^17^ This led Weizenbaum to warn that human engagement with even limited technologies could appear less than fully rational.^17^ The so-called ELIZA effect describes our tendency to ascribe to computers human traits and intentions which we may know they lack.

Many consumer chatbots, such as in banking or telecommunications, are designed to enable human users to efficiently find information or services. In doing this, they may tailor user responses to a history of use to increase search accuracy. Still, the style of interaction here can be relatively simple. In comparison, some chatbots have more ambitious features. For example, Microsoft's Xiaoice, which has over 660 million users, is a social agent ‘with a personality modelled on that of a teenage girl, and [has] a dauntingly precocious skill set’.^20^

Chatbots simulate human conversation by collecting data and finding and projecting patterns. Simple chatbots are often *rule-based*^21^ and follow pre-programmed decision trees or simple ‘If-Then-Else’ rules. Conversational agents with greater flexibility may rely on AI and ML techniques. Some of these employ deep learning neural networks,^22,23^ and some require the collection and storage of large amounts of user data to operate.

Such chatbots may sometimes loosely be said to ‘understand’ or at least to predict and respond to user needs. That chatbots can generate affective responses from users is sometimes regarded as desirable. Microsoft, for example, suggest that ‘[s]ocial chatbots’ appeal lies not only in their ability to respond to users’ diverse requests, but also in being able to establish an emotional connection with users’ and so ‘better understand them and therefore help them over a long period of time’.^24^

In the 1950s, Alan Turing proposed a procedure for determining whether a computer could exhibit human intelligence. Roughly speaking, the Turing Test involves testing whether a device hidden to a person can convince that person that they are interacting with another human being rather than a machine.^25,26^ When chatbots are sufficiently adept, it can seem to the user that they are actually dealing with another human. To avoid this problematic confusion, many current public-facing chatbots reveal at the outset that they are not people. Nonetheless, some other chatbots may not make it particularly clear to users that they are just digital machines.^27^

## Chatbots for mental health: some benefits and challenges

As chatbots proliferated, they moved into the field of mental health. In this field, some chatbots (like many consumer chatbots) are largely a conduit to a human professional.^a^ However, many chatbots, like the popular Woebot,^28^ also provide mental health assistance to users, such as advice and exercises based on cognitive behavioural therapy. These chatbots can often be accessed by anyone who can download an app to a smartphone. Mental health chatbots may also be used by health professionals as an online element of therapies or patient monitoring.^29,30^ Some chatbots, like Replika,^31^ are capable of emulating emotion and psychological connections.

Chatbots sometimes have simple interfaces that provide conversational search engines for digital mental health therapy content.^32^ Whilst chatbots cannot satisfactorily replicate psychotherapeutic dialogue, they can now maintain conversation beyond simple, single linguistic outputs. Some chatbots can guide users through exercises on mental health apps, such as in the examples of Wysa and Tess.^32^ Other chatbots can initiate welcoming chats with clients waiting to see a human therapist via online mental health portals such as e-headspace.^33^ Information useful for therapists can be gathered when chatbots ask clients questions, sometimes using NLP to summarise clients’ responses.^34^

Chatbots have begun to flourish in the digital mental health space partly because they apparently offer certain benefits and advantages. For example, chatbots can perhaps make mental health services and support more accessible for many individuals. They can also run day and night and do not require salaries or sick leave, although they require humans to monitor and update them. When chatbots malfunction, they can be upgraded or switched off. These features have led to interest in chatbots from businesses and organisations and from those who want to expand the supply of mental health assistance amid growing need and insufficient services, further exacerbated by the COVID-19 pandemic.^35–37^

Research shows that in embarrassing or stigmatising circumstances, chatbots may sometimes be preferred to human contact and conversation.^38^ Like Weizenbaum's assistant, users may be more likely to disclose emotional and factual information to a chatbot than to a human. Furthermore, the fact that chatbots may potentially engender emotional connections may be viewed as a potential advantage for socially isolated individuals. We know that in healthcare, a perceived relationship of trust and mutual understanding can be vital for successful therapy.^39^ Chatbots that facilitate emotional connections and that promote trust^40^ and understanding may therefore benefit a range of user groups.

However, some research indicates barriers to using chatbots for mental health. Barriers include privacy concerns, financial constraints, scepticism and reduced willingness among potential users from lower socioeconomic backgrounds.^41^ Some studies have found that the main barriers to chatbot use among adolescents are stigma, embarrassment, poor mental health literacy and preferences for self-reliance.^42,43^ A large-scale survey warned that the current mental health app landscape tends to over-medicalise distress and over-emphasise ‘individual responsibility for mental well-being’.^44^

For chatbot design and use to be successful, users must be able to trust that the technology will meet their needs safely and effectively and that developers are responsive to problems and user feedback.^45^ This point is underscored in the examples of various failed chatbots featured in Table 1. The table illustrates ethical issues caused by chatbots designed to provide, respectively, legal assistance, news, weather and general conversation. As the table shows, these chatbots were the cause of various ethical problems, such as being offensive and causing emotional harm (Lawbot and Tay), misunderstanding user questions (Poncho) and failing to deliver the service and benefits that were promised (newsbots). Mental health chatbots raise similar and also further ethical issues in an especially acute manner, in virtue of the vulnerability of the individuals they are designed to assist. In the next section, we shall see how mental health chatbots present a range of ethical challenges that require careful attention.

**Table 1.** Examples of failed chatbots and reasons behind failure.

**Chatbot**	**Reasons for Failure**
Lawbot: created by Cambridge University students to help victims of sexual assault navigate the legal system^39^	Emotionally insensitive Overly strict checklist to determine what a crime is Can discourage users from seeking help Directs users to local police station but not to support services^40^
Newsbots: In 2016, several news agencies sought to create bots that personalised content and opened up new audiences^38^	Significant resources required for maintenance Did not sync with existing formats, delivery or distribution of news content Lacked sophistication to personalise content effectively Minimal input from journalists during development^38^
Poncho: Weather chatbot using Facebook Messenger with a sassy cartoon cat as the front^41,43^	Sending users unrelated information Not understanding words it should, e.g. ‘weekend’ ^41,43^
Tay: Microsoft chatbot trained via crowdsourced input on Twitter^44^	Shut down after 24 h for producing racist, sexist, anti-Semitic tweets Public able to influence outputs as minimal human supervision provided^44^

## Ethical framework: five principles

Although there is some ethical discussion relevant to mental health chatbots in the literature,^42^ ethical evaluation of such chatbots is still relatively limited.^32^ To make better sense of the ethical issues and to help guide designers, purveyers and practitioners, it makes sense to draw on existing ethical approaches or frameworks. In our view, the five-principle framework outlined by Floridi and Cowls^46^ is particularly useful. In this section, we briefly explain the five-principle ethical framework. We apply the framework to mental health chatbots in the subsequent section.

The five key principles in this framework are (a) non-maleficence, (b) beneficence, (c) respect for autonomy, (d) justice and (e) explicability.^46^ The first four principles are drawn from medical ethics.^12^ The last principle, explicability, has been added because of the special nature of digital technologies such as AI. Explicability is composed of two sub-principles: transparency and accountability. Transparency is important because the ways in which intelligent digital technologies work are often unknown to users (and sometimes even to experts). Accountability is important in part because it can be unclear who is ethically and legally liable for adverse outcomes of intelligent technologies.^46^ Each of the five principles gives a distinctive form of ethical guidance, which we describe in Table 2. The principles are non-absolute but guiding *prima facie* rules for those who design, implement, research or oversee digital technologies.

**Table 2.** Five key ethics principles for mental health chatbots.^46^

**AI Ethics Principle**	** Ethical Requirements**
Non-maleficence	Avoid causing physical, social or mental harm to users
Beneficence	Ensure that interventions do good or provide real benefit to users
Respect for autonomy	Respect users’ values and choices
Justice	Treat users without unfair bias, discrimination or inequity
Explicability	Provide to users sufficient *transparency* about the nature and effects of the technology and be *accountable* for its design and deployment

To be clear, the field of AI ethics has identified many *other* ethical principles relevant to intelligent technologies including chatbots, including safety, solidarity, trust, responsibility and dignity.^47^ However, the five-principle approach has several advantages. First, some of these other principles can be subsumed under the five-principle framework. For example, safety is covered by the principle of non-maleficence, and responsibility is covered by the principle of accountability.

Second, a longer list of overlapping principles can cause confusion and reduce the effectiveness and practicality of a more succinct principle set that is relatively easily remembered and understood. Third, the five principles, with the exception of explicability, have a long track record of usefulness. The principles of non-maleficence, beneficence, respect for autonomy and justice comprise the classic ethical framework introduced in the 1970s by the bioethicists Tom Beauchamp and James Childress.^2,48^ These principles are well accepted in healthcare, whereas other ethical principles (e.g. solidarity and dignity) are less well-known and sometimes more contentious.

It is important to note that while the principles can apply to a range of parties, including those who design and market the technologies, they can apply somewhat differently to mental health practitioners who deploy the chatbots. Mental health practitioners, of course, have especially exacting responsibilities to patients, stemming from their professional roles as health carers. There is thus a contextual element to precisely determine how the principles work in practice. No *prima facie* ethical principle can fully specify how it should be applied in all situations. Nonetheless, as AI ethics scholars have argued, the five principles are still helpfully action-guiding for a range of parties, including technologists.

They are also helpful for those who *research* chatbots for and with people who have mental illness. Here again, context can affect the precise application of the principles. For example, research ethics in healthcare recognises that researchers may balance potential harm to participants against potential benefits to society (e.g. future patients)^49^; in contrast, medical practitioners not involved in research should ordinarily pioritise their current patients’ interests over social benefits in their provision of health services (although they are sometimes required to take social implications into account). This example illustrates how the principles of non-maleficence and beneficence operate somewhat differently in different contexts and require judgement in their application. Again, this need for judgement goes for the other principles in the framework.

## Issues with mental health chatbots: applying the five-principle framework

Guided by these ethical principles, we now identify and discuss four ethically important issues for chatbots in mental health care: human involvement, evidence base, collection and use of data and unexpected disclosure of crimes. Although this list is not exhaustive, it contains key moral considerations and allows us to demonstrate the need for ethical thinking about chatbot use. It also serves to illustrate how the five-principle framework can be used. In the subsequent section, we make some recommendations for addressing the sorts of ethical issues we discuss below.

### Human involvement

To operate successfully and continuously, chatbots require human supervision. As chatbots learn and develop, they may acquire glitches and fail in various ways (Table 1). Thus, human supervision is required to ensure that chatbots operate as desired. Yet, adequate supervision is not always achieved, and this creates the potential for harm. At the same time, chatbot moderation can put pressure on service providers to increase multitasking and workloads in collecting, inputting, organising and constantly updating digital materials, which, paradoxically, may reduce time for teamwork and face-to-face engagement. Further risks arise if there is a power outage that prevents mental health chatbots from providing services.

The complete or relative absence of trained human supervisors from the chatbot environment can undermine the role of expert professionals. Mental health chatbots that provide an automated service are still far from being able to recreate the rich therapeutic alliance^2^ that can exist between patients and human professionals, notwithstanding their efforts to mirror real-life interactions. Though remarkable in its way, ELIZA was never able to substitute for a human therapist and the broad range of skills they possess. The same drawback also applies to more sophisticated, contemporary mental health chatbots, such as those that use AI and NLP and far exceed ELIZA in ‘intelligence’ and learning ability. Recent work, however, is starting to examine if and how a version of the therapeutic alliance, so central to traditional psychotherapy, can be partly emulated or fostered by mental health apps and chatbots.^50,51^

But although increasing personalisation is possible (e.g. different tips/strategies for depression versus anxiety), the support provided by many chatbots at this point in time is still relatively generic and in some ways resembles self-help books. Current chatbots cannot grasp the nuances of social, psychological and biological factors that feed into mental health difficulties. As the popular Woebot warns: ‘As smart as I may seem, I’m not capable of really understanding what you need’.^52^

The explosion of digital technology in health and social services is premised on the reasonable idea that some forms of automation and digital communication could assist with care. One common aim of new technology, such as artificial intelligence, is to break down tasks into individual components that can be repetitively undertaken. However, genuine, comprehensive care is not fully reducible to these tasks, since care also has a rich emotional and social dimension.^53^ Chatbots are not capable of genuine empathy or of tailoring responses to reflect human emotions, and this comparative lack of affective skill may compromise engagement.^42^ Although affective computing is creating systems that can recognise and simulate human emotions,^54,55^ these systems still cannot match the capacities of human therapists.

What does this mean in terms of our chosen ethical framework? The above considerations illustrate ways in which chatbots run some risk of failing to accord with *beneficence* and *non-maleficence*. Because they cannot fully replicate the range of skills and the affective dimensions of a human therapist and because they cannot entirely replace the practitioner–client therapeutic alliance, chatbots may potentially cause harm to some people and thus not align with the principle of non-maleficence. For the same reason, chatbots may also fail to provide the benefits for mental well-being which are intended, thereby not meeting the requirement of beneficence.

However, if mental health chatbots can offer some semblance of an effective therapeutic alliance and/or augment the human–client relationship without causing harm, then they may respect the principles of beneficence and non-maleficence after all. Whether or not this occurs will depend on various factors, such as the nature and competence of the chatbot, the feelings and attitudes of the clients who interact with it, the level of technical support provided and the reliability of the technology and the involvement and role of mental health practitioners.

Where mental health chatbots (or particular instances of them) pose risks of some harm but also promise some degree of benefit, judgement must be used to balance the principles of non-maleficence and beneficence. As we noted earlier, each of the five principles is a *prima facie* rather than absolute principle, and the principles must often be carefully weighed against one another when they point in different directions. For example, if the only option was to offer a client a mental health chatbot due to long waiting lists for practitioners and if that chatbot had the potential to offer some temporary assistance despite carrying certain risks of harm, then it may be judged that beneficence overrides non-maleficence, at least in that specific context. In other cases where the facts are different, non-maleficence may trump beneficence.

### Evidence base

More general ‘mental health’ apps that purport to assist with anxiety, depression and other conditions have been used with varying levels of success. Leigh and Flatt characterise the wide range of mental health apps as suffering from a ‘frequent lack of an underlying evidence base, a lack of scientific credibility and subsequent limited clinical effectiveness’.^56^ There are clear risks with hyping technology, especially for disadvantaged people and without a commensurate evidence base to justify the enthusiasm.^57^ These risks appear at both the individual and population levels, from shaping individual users’ preferences and expectations about service provision to altering how national research funding is distributed. An insufficient evidence base for the deployment of chatbots creates risks that are even more acute for users already suffering various mental health problems.

At present, the evidence base for various mental health chatbots is just getting established. Consequently, there can be uncertainty over whether existing chatbots meet the requirements of *beneficence*. Furthermore, rolling out chatbots may deflect people from essential mental health services and encourage governments and other providers to substitute human for automated care. When such chatbots lack a strong evidence base, this may lead to avoidable harm to people with mental health concerns and thus fail to meet the principle of *non-maleficence*.

We should stress that it is not possible to say precisely when beneficence and non-maleficence will support or oppose the use of a mental health chatbot, for that will depend on the context and circumstances. It will depend, for example, on the relative degree of harm and benefit involved and our knowledge of their probabilities. Uncertainties, after all, are commonplace in healthcare and in regard to emerging technologies. What the principles tell us to do, however, is to make the best judgement we can of the degrees and probabilities of harms and benefits from interventions and to exercise judgement in how we weigh them up to reach conclusions. For instance, if it is likely that a chatbot carries risks of considerable rather than minor harm, the principles will suggest that it is necessary, before chatbot implementation, to have a firmer evidence base that the technology can bring benefits substantial enough to outweigh the risks.

In addition to beneficence and non-maleficence, the above considerations also bring into play the principle of *justice*. An insufficient evidence base, especially for higher stakes interventions, amplifies risks for users with mental health problems. People with mental illness are already often worse off than others: not only do such people suffer from the effects of the illness, they may also have more trouble keeping and finding employment, find themselves subject to social stigmas and isolation and so on. There is thus a risk of violating the principle of justice by exacerbating their problems with promising but poorly tested technologies. Such an amplification of inequity in society is *prima facie* unfair as well as maleficent. Furthermore, justice may be violated if chatbots which lack evidential support are used to replace investment in and access to mental healthcare provided by human professionals.

### Data Collection, Storage and Use

Some (though not all) chatbots collect large amounts of data about people, including data useful for commercial purposes or government intervention (which may sometimes be authorised, e.g. for people at risk of harming themselves or others). Chatbots are frequently trained on existing data, such as data arising from client interaction with service providers. The specific data used shapes those chatbots’ responses. When data sets are not sufficiently comprehensive or representative of the target group, unintended biases may occur. Some AI applications have been severely criticised for producing biases that harm or discriminate against certain groups and individuals.^58^ High profile examples include facial (mis)recognition and recidivism prediction based on ML models.^59,60^ Comparably, trained mental health chatbots might thus reveal biases against people with certain features, such as when they fail to provide correct information to those particular individuals even though they are reliable overall. Clearly, this outcome may transgress the principle of *justice*.

Further questions concern what data is collected, how data is stored (e.g. on a company-based server like Amazon's versus more localised storage), where data is used and how it is linked to other data.^61^ Raw chat data, metadata and even client use behaviour can be tracked and linked with other online behavioural data. Anonymised data can be de-anonymised by data triangulation to reveal people's identities. Data security issues can arise from the risk of data related to mental health being leaked or hacked into by cybercriminals. Any resulting privacy loss can result in mental harm and reduced control over personal information.

Preventing or not providing control over personal information can breach the principle of *respect for autonomy*. Respect for autonomy involves respect for a person's values (e.g. their interests in privacy) and their ability to make decisions based on those values. Obtaining personal and sensitive information from clients with mental health issues will always be an ethically laden business. Respect for autonomy generally requires gaining fully informed consent from individuals before such information is taken and used.

Where a chatbot user is not given sufficient information about what data is collected, how it is used and the risks that such use may generate, the principle of *explicability* and its sub-principle of *transparency* also come into play. Transparency is ethically important because people in general want to know how their data is being managed and what its implications are, including the potential for harm. However, many people are not aware of the ways in which new technologies can harvest and recombine data to make predictions about their identity and behaviour. As noted, such predictions can sometimes be biased against individuals from certain populations.^59,60^ These technologies may thus require special explanations of the risks and benefits of data collection and use, including being given clear information about the likelihood that their data, anonymised or not, could be passed or sold to third parties. These are important reasons why the principle of explicability/transparency—a principle developed as a result of the complex, unfamiliar and autonomous nature of intelligent machines—is a useful addition to the ethical framework for mental health chatbots.

### Unexpected Disclosure of Crimes

The issue of unexpected personal disclosures^15^ is typically overlooked for chatbots, yet it too raises important questions. Consider a user apparently disclosing crimes like child abuse or domestic violence.^62^ As we observed earlier, users can form quasi-relationships with machines,^63^ and this could promote the revelation of information that, if disclosed to a human, might entail ethical or legal duties to report.

It may sometimes be unclear whether such a duty applies to unsupervised chatbots. In some jurisdictions, mental health practitioners (and other professionals) may be legally required to report suspected abuse. But it may be less clear whether a company that provides the technology or obtains and keeps the data has such a legal duty. Principles of *justice* and *beneficence* suggest at least an *ethical* duty of this kind where the disclosure is credible. However, the issue is fraught since it may be unclear whether reporting might exacerbate harms to people with mental health issues and it can be hard to determine what degree of certainty is required to justify it. Here, the principle of justice may conflict with the principle of *non-malefience*. On the one hand, failures to report may lead to serious harm to innocent victims; on the other hand, mistaken reports may effectively cause injustice. Mistaken reports may also undermine trust in chatbot use, which could reduce their overall benefits.

The question of reporting apparent disclosure of crime is, then, yet another occasion on which the ethical principles need to be carefully considered, weighed and balanced to determine the right or best course of action. Even so, the principles give us direction on how to proceed in making such decisions. Clarification about what the law requires or might require could also help here, and future research could beneficially explore the legal ramifications of criminal disclosure across various jurisdictions.

## Recommendations

We are now in a position to offer some recommendations for the design and use of chatbots for mental health. Whether and how a chatbot should be developed and implemented requires an overall ethical evaluation that can be made on the basis of conformity with our *prima facie* ethical principles, suitably interpreted and weighed. In addition to complying with existing law, those responsible for chatbot design and deployment, we suggest, should meet duties of non-maleficence, beneficence, autonomy, justice and explicability (transparency and accountability).

The sub-principle of *accountability*, which we have not yet discussed, refers to the roles and duties of responsible parties to act ethically in their handling of technology. In effect, this means being responsive to the other ethical principles in the framework and establishing appropriate mechanisms and procedures for upholding them. Accountability is thus a means of ensuring that the design and use of chatbots brings benefit, avoids or minimises harm, respects autonomy, remains transparent and is just or fair. To meet the ethical principles, relevant parties (e.g. mental health practitioners and chatbot purveyers) should take the following steps.

### Recommendation 1: weigh risk and benefit

In this step, relevant parties should clearly define the problem they wish to solve and the purpose they want to aim at to ensure the specific chatbot may justifiably be developed in the first place.^64,65^ Sometimes, the risks will be too high or the benefits too low to justify (partially) replacing human therapists with chatbots which cannot empathise or provide comprehensive mental healthcare and which might deflect some people from seeking human care that would be better for them.^2^

If the use of a chatbot is presumptively justified, the above ethical principles should be used to determine how best to develop and implement them throughout the technology pipeline. When existing systems are repurposed or retired, an evaluative process of weighing risk and benefit should be repeated. It might also be worth considering patient and public involvement in mental health chatbot development and research^66,67^ to anticipate and respond to risks, and to maximise the benefits, of chatbots for end-users.

### Recommendation 2: seek and disclose evidential support

As we saw, having a sufficient evidence base^68^ for providing services for disadvantaged people is required by principles of non-maleficence, beneficence and justice. Although their speed and scalability may be tempting, the use of chatbots requires an evidence-based approach. Where the stakes are particularly high (e.g. highly at-risk people with psychological problems), this may require more substantial evidential support, such as well-conducted clinical trials. In less risky situations (e.g. when people are mildly unwell), less evidential support may be acceptable. The degree of evidential support should also be transparently disclosed to respect user autonomy to engage or decline chatbot assistance. While a lack of robust evidence does not imply that chatbots lack value, it does require caution in recommending chatbots and warrants further research into their benefits and risks.^69^

### Recommendation 3: approach data collection/use appropriately

Because collection, storage and use of personal data create risks, the relevant facts must be made transparent to users in order to promote their trust and respect their autonomy. Special attention must be paid to ensuring transparency and adequate understanding for users with mental health issues (or other vulnerabilities) that could impair their understanding. Chatbot developers and owners should ensure that training data is sufficiently representative to mitigate injustice against individuals and groups. Data use also raises legal and ethical questions about privacy. Systems must ensure the security of data to avoid maleficence and disrespect for autonomy. Here, experts in consumer protection, privacy protection and security of data storage may offer important advice.

Data protection laws in many jurisdictions place strict limits on what data (particularly sensitive personal data) can be collected and how it is stored and re-used.^70,71^ Data-related obligations are increasingly demanded by law, such as the EU's General Consumer Data Regulation (GDPR). These demands may increase as societies recognise the implications of big data and the power it lends organisations.^72^ After chatbot retirement, owners should determine how data will be safely stored or destroyed and users should be adequately notified.

### Recommendation 4: consider possible disclosure of crimes

A failure to report to authorities may create legal risks, and reporting crimes when others may be at imminent risk is also a *prima facie* ethical duty of beneficence and justice. Nonetheless, reporting too carries dangers. Theoretically, reporting could be done automatically or else with a human in the loop. One option is to develop a system that scans all user input for problematic content (e.g. using keyword analysis or more sophisticated NLP detection techniques for determining concerning terms/phrases). If a portion of content were to signal an emergency situation or deemed to be beyond the chatbot's purview, then it would be automatically passed on to a human content moderator with sufficient experience who could then make the decision to report or not based on an ethical assessment of the situation. Either way, those utilising chatbots need to be aware of possible legal implications and liabilities.

Accountability might require other steps to be taken. According to Duggal and colleagues, a robust regulatory framework in digital mental health contexts will only emerge when service users, patients, practitioners and providers collaborate to design a ‘forward thinking, future proof, and credible regulatory framework that can be trusted by all parties’.^67^ Without such accountability, there is a higher risk of costly technologies being introduced without thoughtful regard for ethical principles like beneficence, non-maleficence, transparency and respect for autonomy. Poor user consultation also increases the likelihood of wasted resources, which is not only a pragmatic consideration for developers but sometimes also a matter of justice.

Deliberative, participatory development may also be important since services and technology tend to emerge from a concentration of power, such as through government agencies, venture capital and Big Tech, universities with large-scale infrastructure for tech development and sizable professional associations. To ensure greater justice and benefit in design, development and regulation, some writers have called for ‘interdisciplinary empirical research on the implications of these technologies that centres the experiences and knowledge of those who will be most affected’.^73^ Such research should preferably accommodate diversity amongst end-users in terms of age, race, gender, socioeconomic status and so forth, as such factors can shape how users experience technologies.

Undertaking genuinely participatory, community-engaged and inclusive development is not straightforward. Design of technology like chatbots that have the potential to both benefit and harm vulnerable groups should be done via careful consultation with the relevant experts and target users and always with the key ethical principles in mind.

## Conclusion

This paper identified and discussed ethical questions raised by emerging mental health chatbots. Chatbots can probably provide benefits for people with mental health concerns, but they also create risks and challenges. The ethical issues we identified involved the replacement of expert humans, having an adequate evidence base, data use and security, and the apparent disclosure of crimes. We discussed how these ethical challenges can be understood and addressed through the five principles of beneficence, non-maleficence, respect for autonomy, justice and explicability (transparency and accountability), noting that the application of such principles, including where they come into apparent conflict with each other, requires contextual judgment. Based on our discussion, we offered several ethical recommendations for those parties who design and deploy chatbots. While we focused on chatbots for mental health, the ethical considerations we discussed also have broad application to chatbots in other situations and contexts, especially where the end-users are particularly vulnerable.

## Notes

a See: “6 ways Head to Health can help you,” Australian Government Department of Health, https://headtohealth.gov.au/; “Woebot Health,” https://woebot.io; “Wysa—Your 4 am friend and AI life coach,” https://www.wysa.io/; “Joyable,” https://joyable.com/; “Talkspace,” https://www.talkspace.com. Accessed 17 December 2022.
